# Supplementary material for: A hierarchical reinforcement learning model explains individual differences in attentional set shifting
Source: Cogn Affect Behav Neurosci. 2024 Sep 23;24(6):1008–22. doi: 10.3758/s13415-024-01223-7 (PMC11525250; doi:10.3758/s13415-024-01223-7)
Supplement: Supplementary file 1 — Supplementary file1 (DOCX 185 KB) [file 13415_2024_1223_MOESM1_ESM.docx]

**
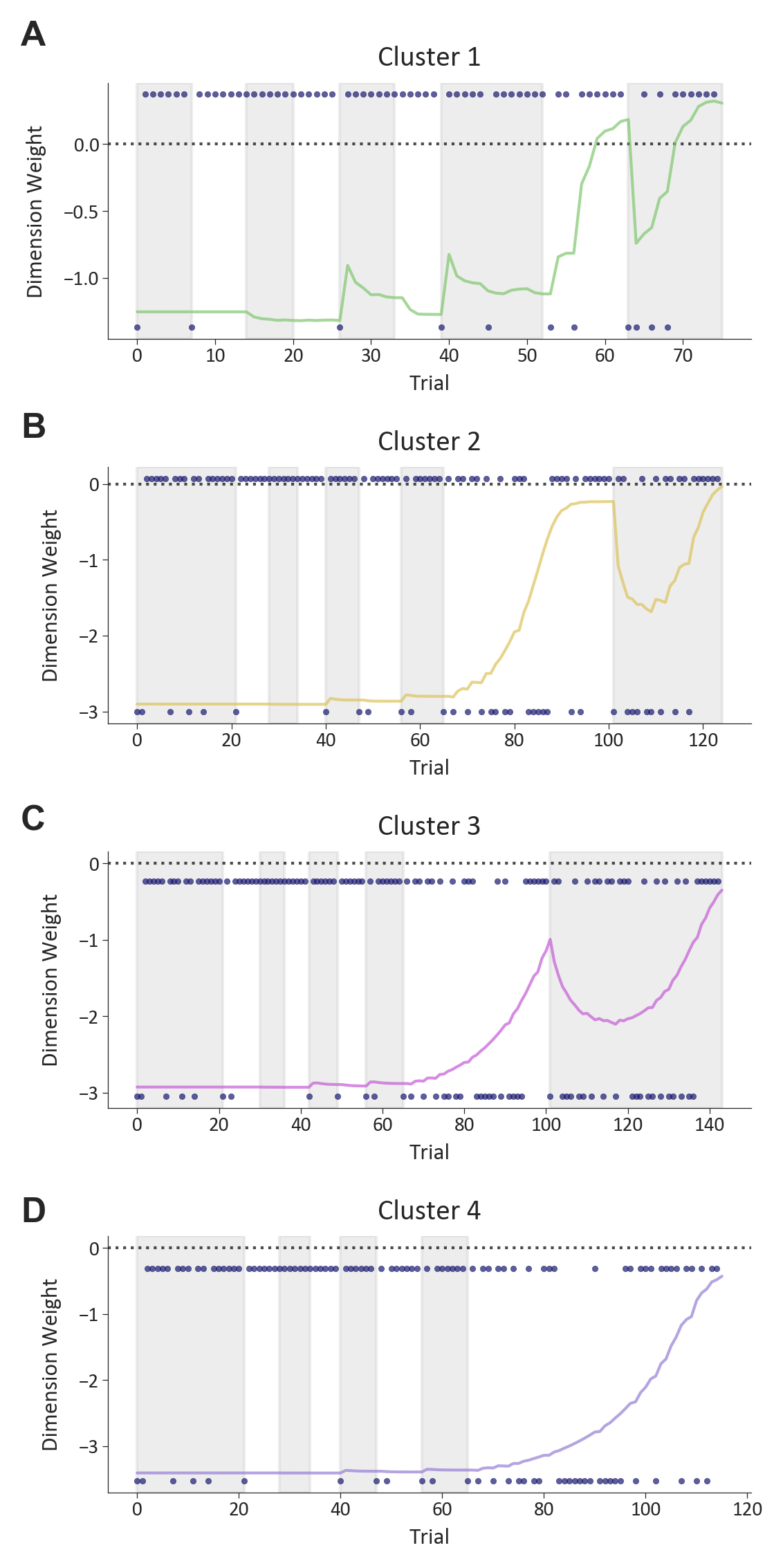
**Supplementary Information

**Figure S1.** Internal model values from simulations. Model simulated dimension weights and rewards for a selected participant from cluster 1 (A), cluster 2 (B), cluster 3 (C) and cluster 4 (D). Lines indicate the dimension weight: when less than 0, participants pay more attention to shapes (the first relevant dimension in these examples), but when more than 0, participants may more attention to lines. Shading indicates successive stages of IED. Blue dots indicate feedback on each trial: those at the top of the panel indicate correct choices, whilst those at the bottom of the panel indicate incorrect choices.

**Table S1.** Range of possible questionnaire scores, along with mean, standard deviation, and median of participant scores. OCI-R: Obsessive Compulsive Inventory-Revised, STAI-S: State Trait Anxiety Inventory – State, STAI-T: State Trait Anxiety Inventory – Trait, SRDS: Self-Rating Depression Scale, SSMS: Short Scales for Measuring Schizotypy.

|  | **Measure** | **Range** | **Mean ± SD, Median** |
| --- | --- | --- | --- |
| **Questionnaire** | OCI-R | 0 - 72 | 14.72 ± 10.93, 12 |
|  | STAI-S | 20 - 80 | 36.68 ± 12.83, 35 |
|  | STAI-T | 20 - 80 | 42.52 ± 14.31, 41 |
|  | SRDS | 20 - 80 | 39.89 ± 10.54, 40 |
|  | SSMS | 0 - 41 | 12.14 ± 7.07, 11 |
